# Attenuating Effect of Peruvian Cocoa Populations on the Acute Asthmatic Response in Brown Norway Rats

**DOI:** 10.3390/nu12082301

**Published:** 2020-07-31

**Authors:** Marta Périz, Francisco J. Pérez-Cano, Trinitat Cambras, Àngels Franch, Ivan Best, Santiago Pastor-Soplin, Margarida Castell, Malén Massot-Cladera

**Affiliations:** 1 Secció de Fisiologia, Departament de Bioquímica i Fisiologia, Facultat de Farmàcia i Ciències de l’Alimentació, Universitat de Barcelona (UB), 08028 Barcelona, Spain; mperiz@ub.edu (M.P.); franciscoperez@ub.edu (F.J.P.-C.); cambras@ub.edu (T.C.); angelsfranch@ub.edu (À.F.); margaridacastell@ub.edu (M.C.); 2 Institut de Recerca en Nutrició i Seguretat Alimentària (INSA-UB), UB, 08921 Santa Coloma de Gramenet, Spain; 3 Programa Cacao, Ingeniería Agroforestal, Facultad de Ciencias Ambientales, Universidad Científica del Sur, Lima 15842, Peru; ibest@usil.edu.pe (I.B.); spastor@ucientifica.edu.pe (S.P.-S.); 4 Unidad de Investigación en Nutrición, Salud, Alimentos Funcionales y Nutracéuticos, Universidad San Ignacio de Loyola, Lima 15024, Peru

**Keywords:** anaphylaxis, asthma, IgE, leukotriene, mast cell protease, methylxanthines, motor activity, polyphenols, temperature, *Theobroma cacao*

## Abstract

Cocoa contains bioactive components, which vary according to genetic and environmental factors. The present study aimed to ascertain the anti-allergic properties of native Peruvian cocoa populations (“Blanco de Piura” or BPC, “Amazonas Peru” or APC, “Criollo de Montaña” or CMC, “Chuncho” or CCC, and an ordinary cocoa or OC). To do so, after an initial in vitro approach, an in vivo study focused on the induction of an anaphylactic response associated with allergic asthma in Brown Norway rats was carried out. Based on their polyphenol content, antioxidant activity and in vitro effects, the APC and CMC were selected to be included in the in vivo study. Cocoa diets were tested in a model of allergic asthma in which anaphylactic response was assessed by changes in body temperature, motor activity and body weight. The concentration of specific immunoglobulin E (IgE), mast cell protease and leukotrienes was also quantified in serum and/or bronchoalveolar lavage fluid. CMC and OC populations exhibited a protective effect on the allergic asthma rat model as evidenced by means of a partial protection against anaphylactic response and, above all, in the synthesis of IgE and the release of mast cell protease.

## 1. Introduction

Allergic asthma is a complex inflammatory disorder characterized by chronic airway inflammation and immune-mediated hypersensitivity reaction [1]. Asthmatic patients often present airflow limitation and suffer from variable respiratory symptoms such as wheezing, shortness of breath, chest tightness and cough [2]. Histologically, the airway of an asthmatic patient is characterized by eosinophils infiltration, degranulated mast cells together with alteration of epithelial cell tight junctions and hyperplasia of goblet cells [3,4]. Although allergic asthma often starts in childhood, its prevalence is also high in adults, and affects around 270 million people worldwide [5]. Peru is one of the countries with the highest prevalence of asthma in Latin America [6,7], where 19.6% of adolescents (13–14 years old) suffer from asthma. However, this disease is underdiagnosed and the prevalence of physician-diagnosed asthma in Peru has been reported to be 33.1% [6].

Asthma therapy includes pharmacological interventions as well as treatment of associated comorbidities and modifiable lifestyle risk factors (e.g., avoidance of tobacco and weight loss). The pharmacological treatment consists of short-acting β_2_-agonists and inhaled corticosteroids, although adherence is often poor. In addition, emerging biological therapies, such as monoclonal antibodies targeting several cytokines, show promising results [2]. Nevertheless, non-pharmacological strategies can reveal some benefits in reducing symptoms and corticosteroid use. In this context, it has been proposed that diet could represent a good complement to controlling allergic asthma disease.

Flavonoids, chemically belonging to the polyphenol class, are a large family of secondary products of plants that contribute to the blue, scarlet and orange colors of their leaves, flowers and fruits. They are found in seeds, nuts, grains and spices and in some derived beverages such as wine, tea, cocoa and beer [8]. Due to their chemical polyphenolic structure, flavonoids have demonstrated antioxidant properties. This is why, in the past few years, flavonoids have emerged as potential therapeutic/coadjuvant agents in several conditions, such as in cardiovascular diseases, in chronic inflammation, in cancer and also in allergies and asthma [8,9,10,11].

The cocoa bean and its derivates (e.g., cocoa powder, chocolate, etc.) have bioactive compounds and, among them, flavonoids [9]. In particular, cocoa contains (+)-catechin and (−)-epicatechin as monomers and procyanidins (from 2 to 10 monomeric units) as polymers [9]. These flavonoids together with other bioactive compounds, such as theobromine and fiber, confer on cocoa immunomodulatory properties both in vitro and in vivo [12,13,14,15,16,17]. In particular, cocoa consumption has previously shown anti-allergic properties in a model of systemic disease [18,19] and oral sensitization [20]. In addition, a protective effect on allergy has also been suggested when considering cocoa consumption in young people [21].

Effects of cocoa intake will vary depending on the amount of flavonoids and other bioactive compounds present in the product. Polyphenolic content of cocoa products varies greatly due to genetic factors, such as the variety or clone their beans come from [22,23]. However, genetics only partially determines the biochemical profile. The phenolic content of cocoa products also varies greatly due to environmental factors such as soil, water, cultivation latitude, management and post-harvest handling of the product (fermentation and drying). Thus, the result is a wide range of regional cocoa populations with singular qualities and quantities of these bioactive compounds [24,25,26].

Based on the above-mentioned, we hypothesized that the cocoa tree (*Theobroma cacao* L.) cultivated under a tropical climate, as in the North of Peru and South of Ecuador, which are considered as being the center of the origin and genetic diversity of cocoa [27,28], may include populations with different biological effects, such as those on the immune system reported so far. Thus, the present study aimed to ascertain the anti-allergic properties of Peruvian cocoa populations, firstly using an in vitro approach to select the most active populations and secondly using an in vivo study focused on the induction of an anaphylactic response associated with allergic asthma in Brown Norway rats.

## 2. Materials and Methods

### 2.1. Cocoa Population Characterization

Pastes made with beans from four Peruvian cocoa populations were used: “Blanco de Piura” (BPC) from the Piura region (latitude/longitude −5.270248, −79.964108), “Amazonas Peru” (APC) from the Amazonas region (−5.737422, −78.431114), “Criollo de Montaña” (CMC) from the Junín region (−11.335774, −74.533181), and “Chuncho” (CCC) from the Cusco region (−12.510664, −73.834577). As reference cocoa, CCN-51 ordinary cocoa paste from the same area as the CCC was included. With the exception of the CCN-51, these are the populations of Peruvian cocoa considered to be fine or flavor cocoa (Article 39, ICA, 2010) [29], and due to their morpho-agronomic and sensory properties, they are best known and characterized for their use in making high quality artisan chocolates [30,31]. The cocoa samples were obtained under prior informed consent (PIC), in agreement and signed with farmers, and in accordance with the Nagoya Protocol spirit of sharing the benefits arising from the utilization of genetic resources [32,33]. BPC comes from a coastal area, facing the Pacific Sea. It develops in a dry and warm environment, but under irrigation and neutral loamy soils. The other cocoa populations are found on the eastern side of the Andes, in the Amazon, and they develop in rain fed on acidic and clay loam soils. The cocoa pastes were made at the place of origin, based on a common protocol. Biochemical analysis, which was performed in triplicate, began with 100% pure cocoa paste.

#### 2.1.1. Phenolic Compounds and Antioxidant Activity

The extraction of bioactive compounds from the different cocoa samples was carried out using the methodology proposed by Pedan et al. [34], with minor modifications. The cocoa paste was heated in a water bath until it reached a liquid state. To remove lipids, 20 mL of each sample was placed in a 250 mL flask and 80 mL of n-hexane was added (5 min at 20 °C) and then centrifuged (2880× *g*, 5 min). The defatting procedure was repeated five times, until the n-hexane extract remained colorless. After drying, 5 g of cocoa powder with an average particle size of less than 100 µm was extracted three times with 15 mL of acetone/water (50/50) (8 min at 50 °C) and then centrifuged (2880× *g*, 5 min). The supernatants obtained in each extraction step were mixed and used to measure the total levels of phenols and flavonoids using the Folin-Ciocalteu method [35] and the aluminum chloride colorimetric method [36], respectively.

In vitro antioxidant capacity was evaluated using the α,α-diphenyl-β-picrylhydrazyl (DPPH) radical scavenging assay [37] and the ferric-reducing/antioxidant power (FRAP) assay [38].

#### 2.1.2. Methylxanthine Quantification

High-performance liquid chromatography (HPLC) determination of theobromine, theophylline, and caffeine was performed according to Srdjenovic et al. [39] with minor modifications. Firstly, an extract was prepared (2.5 g of cocoa powder with 10 mL of water) and then incubated in an ultrasonic bath (30 min at 60 °C). After centrifuging (4000× *g*, 10 min, 20 °C), 10 mL of the supernatants was purified using solid phase separation (SPE) with a Supelclean LC-18 SPE cartridge (Sigma-Aldrich, St. Louis, USA). Samples were run on a Chromaster 600 HPLC with a diode array detector (Hitachi, Tokyo, Japan), an autosampler and a C8 reverse-phase column (5 µm particle size, i.d. 4.6 × 150 mm). The mobile phase consisted of water-tetrahydrofuran (0.1% in water, pH 8)—acetonitrile (90:10, *v/v*); the run time was 8 min with a flow rate of 0.8 mL/min. Detection was performed at 273 nm using a photodiode array detector.

### 2.2. Animals

Four-week-old female Brown Norway rats were obtained from Envigo (Huntingdon, UK) and housed (3 rats per cage) in the animal facilities at the Faculty of Pharmacy and Food Science (University of Barcelona) in polycarbonate cages containing bedding of large fibrous particles (Souralit 1035, Bobadeb S.L., Santo Domingo de la Calzada, Spain) under controlled conditions of temperature and humidity in a 12:12 h light/dark cycle. The animals remained in quarantine for 1 week before experiments began.

All experimental procedures were conducted in accordance with the institutional guidelines for the Care and Use of Laboratory Animals and were approved by the Ethical Committee for Animal Experimentation of the University of Barcelona and the Catalonia Government (CEEA/UB ref. 414/16 and DAAM 9351, respectively), in full compliance with national legislation following the EU-Directive 2010/63/EU for the protection of animals used for scientific purposes.

### 2.3. In vitro Study

#### 2.3.1. Peritoneal Macrophages and Lymphocytes Culture

Peritoneal macrophages and spleen mononuclear cells were obtained from six healthy rats under anesthesia with ketamine (90 mg/kg) (Merial Laboratories S.A, Barcelona, Spain) and xylazine (10 mg/kg) (Bayer A.G, Leverkusen, Germany).

Peritoneal macrophages were collected as previously described [40]. Briefly, after the injection of 40 mL of ice-cold sterile phosphate buffered saline (PBS, pH 7.2) into the peritoneal cavity, a 1 min massage was performed, and cell suspension was aspirated and centrifuged (538× *g*, 10 min, 4 °C). After removing possible erythrocytes by osmotic lysis (ammonium chloride), cells were resuspended with cold Roswell Park Memorial Institute (RPMI) medium without phenol red (Merck, Madrid, Spain), supplemented with 10% heat-inactivated fetal bovine serum (FBS), 100 IU/mL streptomycin-penicillin, 2 mM L-glutamine and 0.05 mM 2-mercaptoethanol (Sigma-Aldrich, Madrid, Spain). Macrophage counts were assessed using a Spincell hematology analyzer (MonLab Laboratories, Barcelona, Spain), properly calibrated for these cells, and were plated (10^6^ cells/mL) at 37 °C overnight. After removing non-attached cells, macrophages were incubated with 10 μg/mL of each of the five cocoa extracts in dimethyl sulfoxide (DMSO) for 2 h. Afterwards, cells were stimulated with 100 ng/mL lipopolysaccharide (LPS) for 6 h. Stimulated macrophages with no cocoa addition were used as control. Cell viability was measured through determination of lactate dehydrogenase (LDH) enzyme released to the medium. Macrophages were also used to establish M1/M2 polarization.

Spleen mononuclear cells were isolated from rat spleens as previously described [41,42]. Firstly, spleen cell suspensions were obtained by passing the tissue through a cell strainer (40 μm, BD Biosciences, Heidelberg, Germany), and then erythrocytes were eliminated by osmotic lysis. A Countess™ Automated Cell Counter (Invitrogen™, Thermo Fisher Scientific, Waltham, MA, USA) was used for cell counting and the assessment of viability. Splenocytes (10^6^ cells/well) were immediately incubated in the presence of 10 μg/mL of each of the five cocoa extracts in DMSO for 2 h. Afterwards, splenocytes were stimulated with 100 ng/mL LPS or remained nonstimulated for 24 h, and then, supernatants from both conditions were collected for TNF-α determination. In parallel, nonstimulated splenocytes were cultured for 96 h, whose supernatants were used to quantify the IgG. Both assays were performed in quadruplicate.

#### 2.3.2. Radical Oxygen Species (ROS) Production

ROS production was quantified in isolated macrophages as previously described [42]. In brief, macrophages were plated (10^5^ cells/well) and allowed to attach overnight. Then, they were washed with warm 1% FBS-supplemented RPMI medium without phenol red. Macrophages were incubated with 20 µM of reduced 2′,7′-dichlorofluorescein diacetate (H_2_DCF-DA) probe (Invitrogen, Paisley, UK) for 30 min at 37 °C. Macrophage-derived ROS oxidized H_2_DCF-DA to a fluorescent compound (20,70-dichlorofluorescein, DCF), which was quantified using the fluorimeter Modulus^®^ Microplate Multimode Reader (excitation 538 nm, emission 485 nm, Turner BioSystems, CA, USA). ROS results are expressed as the time course from 0 to 130 min and also as the area under the curve (AUC) of this period of time.

#### 2.3.3. M1 and M2 Characterization

After cocoa incubation and LPS stimulation, the M1 and M2 phenotype of macrophages were established by the exclusive expression of the molecules CD86 and CD206, respectively [43,44]. For M1 phenotype, an anti-rat CD86 conjugated to phycoerythrin (Biolegend, San Diego, CA, USA) was applied as in previous studies. For M2 phenotype, a primary rabbit polyclonal antibody (Ab) to mannose receptor (CD206) (Abcam plc, Cambridge, UK) was used, followed by blocking nonspecific signals, and a secondary Ab conjugated to Alexa-Fluor-647 (Abcam plc), as in previous studies [45]. A negative control using isotype-matched Ab was included for each sample. Data were acquired with a Gallios™ Cytometer (Beckman Coulter, Miami, FL, USA) in the Flow Cytometry Unit of the Scientific and Technological Centers of the UB (CCiTUB) and analyzed with FlowJo v.10 software (Tree Star, Inc., Ashland, OR, USA). Results are expressed as percentages of positive cells in the macrophage population previously selected according to their forward-scatter (*FSC*) and side-scatter (*SSC*) characteristics.

#### 2.3.4. IgG and Tumor Necrosis Factor (TNF)-α Quantification by ELISA

TNF-α were quantified in the 24 h supernatants by stimulated splenocytes using Opt-EIA-set (BD Biosciences), as in previous studies [46]. IgG were quantified in 96 h supernatants by nonstimulated splenocytes with an enzyme-linked immunosorbent assay (ELISA) following the manufacturer’s instructions (BD Biosciences), as previously described [46]. In both cases, absorbance was measured in a microplate photometer (LabSystems Multiskan) and analyzed using ASCENT version 2.6 software (Thermo Fisher Scientific, Waltham, MA, USA). TNF-α and IgG results are shown as percentage with respect to the control condition (without cocoa), which was considered as 100%.

### 2.4. In vivo Study

According to their polyphenol content, antioxidant activity and their in vitro effect on macrophages and splenocytes, two populations of Peruvian cocoa were selected: “Amazonas Peru” cocoa (APC) and “Criollo de Montaña” cocoa (CMC). The in vivo effects of these two populations were then established in a model of allergy in rats. The ordinary cocoa (OC) was also included to be considered as a reference cocoa.

#### 2.4.1. Diets and Animal Groups

Four diets were elaborated: a standard diet based on the AIN-93M diet (Envigo) and three diets in which 90% of powdered AIN-93M was mixed with 10% of cocoa paste (OC, APC or CMC) previously pulverized. The mixture was pelletized and subsequently dried in a 40 °C oven for 36 h. Once dried, the pelleted diet was vacuum-packed to prevent oxidation and contamination and stored at 4 °C until used.

The animals were randomized into five experimental groups (*N* = 9 animals/group): the healthy reference group (REF) and asthmatic group (A) were both fed with the standard diet, and the three asthmatic groups received the OC, APC and CMC diets, respectively (CC, APC and CMC groups). The animals had free access to the experimental diet and water. The body weight and food and water intake were monitored every 2–3 days throughout the study. The Appraising Project Office’s program from the Universidad Miguel Hernández de Elche (Alicante, Spain) was used to calculate the minimum number of animals providing statistically significant differences among groups, assuming that there is no dropout rate and type I error of 0.05 (two-sided). In addition, the sample size was adjusted following the University Ethical Committee guidelines and to apply the three Rs rule for experimenting in animals.

#### 2.4.2. Sensitization and Induction of an Anaphylactic Response

At 1 week after the beginning of the experimental diet, asthma was induced using ovalbumin (OVA) as allergen, as previously described [47]. Briefly, on day 0, rats were firstly sensitized via intraperitoneal (i.p.) with 500 µL of a suspension containing 50 µg of OVA (grade V, Sigma-Aldrich, Madrid, Spain), 20 mg of alum (Imject^®^; Pierce, IL, USA) and 50 ng of *Bordetella pertussis* toxin (Sigma-Aldrich) and boosted a week later with 50 µg of OVA in 20 mg of alum (i.p.). A parallel group of non-sensitized rats (age and sex matched) was included.

At day 28, between 10 a.m. and 1 p.m., all rats received an intranasal (i.n) challenge with 300 µL of an OVA solution (50 mg/mL). Anaphylactic response was accurately assessed by changes in motor activity, body temperature, body weight and plasma protease concentration. Anti-OVA IgE was quantified in blood and bronchoalveolar lavage fluid (BALF) samples obtained 24 h later.

#### 2.4.3. Body Temperature Monitoring

In order to monitor the body temperature, data loggers (Thermochron^®^, iButton type DS1921H-FS with a resolution of 0.125 °C) were used. For this, 1 week before the i.n. challenge, a logger was intraperitoneally implanted in each rat under isoflurane (Isoflo^®^, ECUPHAR, Barcelona, Spain) anesthesia (4–5% in the induction, 1–2% in the maintenance with an oxygen flow of 0.5–1.0 L/min). Meloxicam (1 mg/kg body weight, subcutaneous route) was administered subcutaneously immediately after the intervention and 24 h later. Animals were then housed in individual cages in an isolated room (see motor activity assessment section). Body temperature was recorded every minute from the night before the challenge (starting at 2 a.m.) until the day after the challenge, when the sensor was removed. The results of body temperature are expressed as the time course of the mean values during the registered period, the mean value every 2 h from 2 h before the challenge to 18 h after the i.n. challenge and as the AUC between 900 and 400 min after the challenge considering changes above 34 °C.

#### 2.4.4. Motor Activity Assessment

The movement of animals housed in individual cages and placed in an isolated room were quantified using an activity meter, as previously performed [47,48]. The activity meters consisted of two infrared beams that crossed perpendicularly 7 cm above the floor of the cage. Every time the animal crossed one beam a count was detected. Number of movements was recorded every minute from 2 days before the i.n. challenge until 18 h after. To summarize the effects of anaphylactic response on motor activity, the total number of movements in the active period of the rats (darkness period from 8 p.m to 8 a.m) was considered, with the exception of the last 2 h in order to avoid the variations in motor activity in anticipation of light due to the normal circadian rhythm. The movements in the dark period before and after the i.n. challenge were also compared.

#### 2.4.5. Sample Collection

One hour after the i.n. challenge, blood samples from the saphenous vein were obtained to quantify plasma mast cell protease.

Twenty-four hours after the i.n. challenge, the rats were anesthetized with ketamine (90 mg/kg) (Merial Laboratories S.A) and xylazine (10 mg/kg) (Bayer A.G). Urine samples obtained by direct puncture of the bladder were kept at −80 °C until quantification of the polyphenol concentration. Blood samples were collected by heart puncture and kept at −20 °C until anti-IgE determination.

#### 2.4.6. Quantification of Plasma Rat Mast Cell Protease II

Plasma samples obtained 1 h after the i.n. challenge were used to quantify rat mast cell protease II (RMCPII) concentration using a commercial ELISA kit (Bionova, Madrid, Spain) following the manufacturer’s instructions. Results are shown as absorbance units obtained from all samples analyzed in the same ELISA plate compared to that produced by asthmatic rats, which are considered as 100%.

#### 2.4.7. Antibody Quantification

Anti-OVA specific IgE antibody isotype in serum and BALF samples were quantified using an antibody-capture ELISA, as previously performed [18,41]. A pool of positive sera was used as standard in each plate. Serum samples were diluted 1/10, whereas BALF samples were processed undiluted. Results are shown as mean percentage compared to the asthmatic group, which are considered as 100%.

IgE concentration in BALF samples was quantified using a sandwich ELISA, as previously described [47]. Results are shown as mean percentage compared to the asthmatic group, which are considered as 100%.

#### 2.4.8. Quantification of Cysteinyl Leukotriene (CysLT)

The concentration of CysLT in BALF was quantified using an Cysteinyl Leukotriene ELISA kit (Enzo Life Sciences Inc., New York, NY, SUA) following manufacturer’s instructions, with a prior extraction of leukotrienes as previously described [47]. Results are shown as the percentage from that produced by asthmatic rats, which are considered as 100%.

#### 2.4.9. Urine Polyphenols

Total phenolic content in urine samples was determined according to Folin–Ciocalteu’s method adapted to a microplate. Briefly, 250 µL of Folin–Ciocalteau’s reagent (Sigma-Aldrich) and 1.25 mL of 20% Na_2_CO_3_ solution were added to 500 µL of diluted urine. After 2 h at room temperature, the absorbance was measured at 765 nm. A standard curve prepared with gallic acid (Sigma-Aldrich) was used.

### 2.5. Statistical Analysis

The Statistical Package for the Social Sciences (SPSS v22.0, IBM, Chicago, IL, USA) was used for statistical analysis. Data were tested for homogeneity of variance and normality distribution using the Levene’s and Shapiro–Wilk tests, respectively. When data was homogeneous and had a normal behavior, a conventional two-way ANOVA test followed by the post hoc Bonferroni and paired t-test were used in order to assess significance for independent and related samples, respectively. Otherwise, the nonparametric Kruskal–Wallis test followed by the post hoc Mann–Whitney U test were performed. Significant differences were established when *p* < 0.05 for the paired t-test, whereas for multiple comparisons, the *p* value was adjusted following Bonferroni correction [49].

To explore the functional correlation between the antibody levels, CysLT concentration, RMCPII production, body temperature and motor activity changes, Spearman’ correlation analyses were performed in all samples grouped together.

## 3. Results

### 3.1. Cocoa Peruvian Populations Characterization

The content of total phenolics, total flavonoids, theobromine and caffeine differed between the five cocoa samples considered (Table 1). The population with the highest content in phenolics and flavonoids was CMC, followed by APC, and CCC in the third place (*p* < 0.005 CMC, APC and CCC vs. OC; *p* < 0.005 APC vs. CMC; *p* < 0.005 CCC vs. BPC). The OC and BPC cocoa pastes contained the lowest levels of both phenolic and flavonoid content. With regard to methylxanthine content (Table 1), the CMC cocoa paste exhibited the highest amounts of theobromine and caffeine (*p* < 0.00001) followed by the CCC population.

With regard to the antioxidant capacity (Table 1), the APC cocoa paste was the one with the highest capacity, followed by the CMC and CCC populations (*p* < 0.01), whereas the BPC had the lowest antioxidant capacity.

### 3.2. In Vitro Effects of Cocoa Peruvian Populations

**Figure 1:**
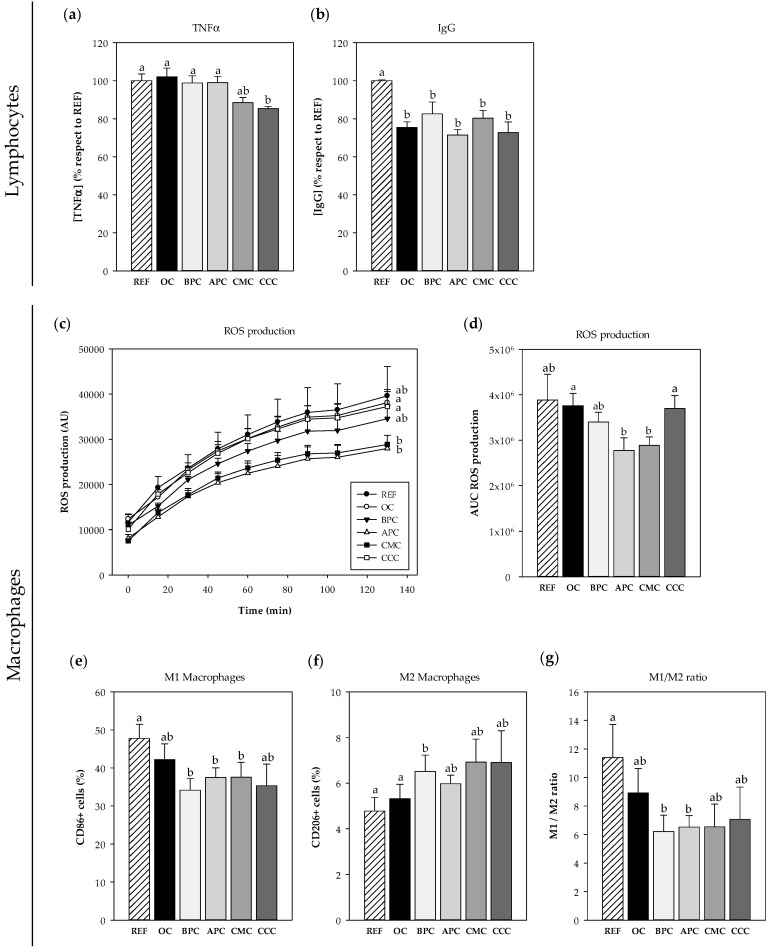
In vitro immunomodulatory effects of the five Peruvian cocoa samples. Effects of cocoa samples on TNF-α (**a**) and spontaneous IgG (**b**) production by splenocytes. Effects of cocoa samples on macrophages: oxygen reactive species (ROS) production (**c**) over time and as area under the curve (AUC) (**d**), and phenotype characterization: M1 (**e**), M2 (**f**) and M1/M2 ratio (**g**). REF: cells with no cocoa; OC: ordinary Peruvian cocoa; BPC: “Blanco de Piura”; APC: “Amazonas Peru” cocoa; CMC: “Criollo de Montaña” cocoa; CCC: “Chuncho” del Cusco. Results are represented as mean ± standard error of the mean (*N* = 6). Values not sharing letters denote significant differences between cocoa samples (*p* < 0.01), while values sharing the same letter did not differ.

An approach to study the immunomodulatory effects of each cocoa population in vitro on spleen lymphocytes and peritoneal macrophages was carried out (Figure 1).

#### 3.2.1. Effects on Spleen Cells

The viability of rat spleen mononuclear cells was not affected by the cocoa addition, which was around 90% in all cases. In these conditions, CMC and CCC cocoa populations were able to prevent the secretion of TNF-α after LPS stimulation (*p* < 0.01) (Figure 1a). Moreover, all cocoa populations lowered spontaneous IgG production compared to the control (*p* < 0.01) (Figure 1b).

#### 3.2.2. Effects on Macrophages

Cell viability of rat macrophages did not decrease after cocoa addition but the ROS production was significantly reduced in cells incubated with both APC and CMC populations throughout the studied period, as observed in the time course (Figure 1c) as well as in the AUC (*p* < 0.01 vs. OC and CCC samples) (Figure 1d). In addition, when analyzing the proportion of M1 (pro-inflammatory) and M2 (anti-inflammatory) macrophages after LPS stimulation, it was observed that three of the samples tested (BPC, APC and CMC) decreased the proportions of M1 cells compared to the stimulated cells with no cocoa (Figure 1e). Moreover, the BPC population was able to significantly increase the proportion of M2 macrophages (*p* < 0.01) (Figure 1e). Overall, although all cocoa samples tended to lower the M1/M2 ratio, only the decrease caused by the BPC and CMC cocoas reached statistical significance (*p* < 0.01) (Figure 1f).

Based on their polyphenol content, antioxidant activity and their in vitro effects on macrophages and splenocytes, the APC and CMC Peruvian cocoa populations were selected to be included in the in vivo study. The OC cocoa was also included to be used as a reference cocoa.

### 3.3. In Vivo Effects of Cocoa Peruvian Populations

#### 3.3.1. Body Weight and Food and Water Intake

At the beginning of the diets, 1 week before asthma induction, animals from all groups had a similar body weight (Table 2). Although the asthmatic animals’ body weight was not significantly modified by either asthma induction or cocoa diets, it tended to be lower than that in the reference animals after the booster and it tended to be even lower with cocoa diets. These changes in body weight were not due to changes in either food or water consumption, which did not vary between diets and groups (Supplementary Tables S1 and S2).

#### 3.3.2. Content of Polyphenols in Urine

**Figure 2:**
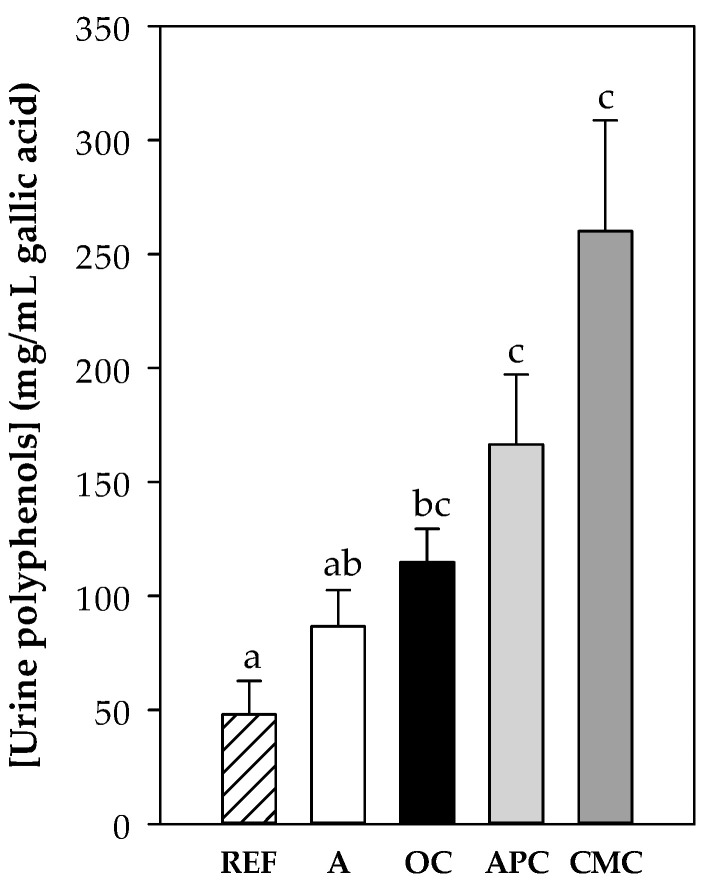
Polyphenol content (expressed as μg/mL gallic acid) in urine samples at the end of the study. REF: healthy reference group fed standard diet; A: asthmatic group fed standard diet OC: asthmatic animals fed with ordinary cocoa-enriched diet; APC: asthmatic animals fed with “Amazonas Peru” cocoa-enriched diet; CMC: asthmatic animals fed with “Criollo de Montaña” cocoa-enriched diet. Results are represented as mean ± standard error of the mean (*N* = 9). Values not sharing letters denote significant differences between groups (*p* < 0.01), while values sharing the same letter did not differ.

Total polyphenol concentration was quantified in urine samples at the end of the study to verify the polyphenol absorption. As expected, urine samples from rats fed the cocoa diets showed higher values than those obtained from the reference and asthmatic rats fed the standard diet (Figure 2). Moreover, the highest polyphenol content was found in CMC- and APC-fed animals’ urine samples, which was the population with the highest flavonoid content (Table 1).

#### 3.3.3. Changes in Body Temperature After i.n. Challenge

**Figure 3:**
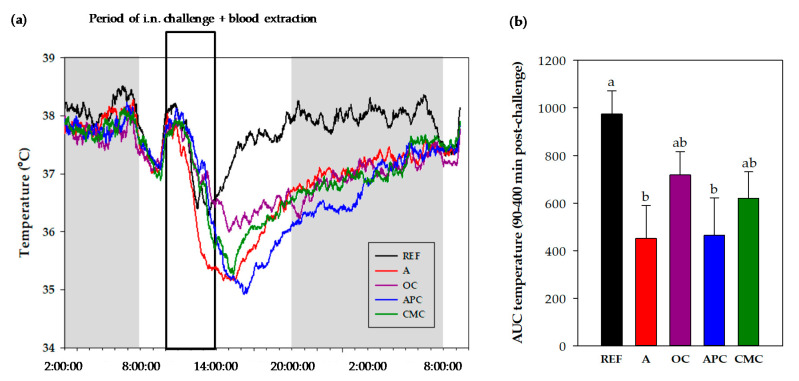
Changes in body temperature. Profile of body temperature from 2 a.m. before intranasal (i.n.) challenge and until 10 a.m the day after. Statistical differences not shown (**a**). Area under the curve (AUC) of body temperature (from 34 °C) in the period comprised between 90 and 400 min after the i.n. challenge (**b**). REF: healthy animals fed standard diet; A: asthmatic animals fed standard diet; OC: asthmatic animals fed with ordinary cocoa-enriched diet; APC: asthmatic animals fed with “Amazonas Peru” cocoa-enriched diet; CMC: asthmatic animals fed with “Criollo de Montaña” cocoa-enriched diet. Results are shown as mean (**a**) or as mean plus standard error of the mean (**b**) (*N* = 9). Values not sharing letters denote significant differences between groups in (**b**) (*p* < 0.01), while values sharing the same letter did not differ.

The body temperature (BT) was registered for each rat from 2 a.m. on the day of the i.n. challenge to 10 a.m. the day after (Figure 3). The mean value profile of BT registered every minute for this period with respect to the hour of the day (independently of the moment of challenge) is shown in Figure 3a, in which the darkness period is represented in gray. Also indicated is the period in which rats received the i.n. challenge and blood was collected. Table 3 summarizes the results of BT adjusted to the moment each rat was challenged and until 18 h after the i.n. challenge.

The i.n. challenge resulted in a reduced BT (Figure 3a and Table 3). However, the profile through the day shows that the REF group was able to recover the BT during the afternoon-evening and remained more or less constant the following night (Figure 3a). On the contrary, the asthmatic rats showed a slower BT recovery than the REF group and achieved the REF animals’ BT at about 8 a.m. the day after the challenge.

Before challenging, the basal BT (mean value for 2 h before challenge) did not differ between groups (Table 3). After the i.n. challenge, all animals reduced their BT and reached the lowest values 2–4 h later. The REF group showed about 1 °C of BT reduction with respect to their own basal BT (*p* < 0.05), in the period between 2 and 6 h after the challenge. The asthmatic group fed a standard diet underwent a decrease of more than 2 °C of BT, which was already detected during the first 2 h after the i.n. challenge. Their BT remained significantly lower during all the period considered when comparing it either to their basal BT (*p* < 0.05) or to the REF animals’ BT at the same time interval (*p* < 0.01).

The OC-fed asthmatic rats showed a BT reduction of about 1.5 °C during the interval of 2–8 h after the i.n. challenge. Their BT was significantly lower compared to their basal BT (*p* < 0.05) and to that in the REF group during the 4–10 h period of time after the challenge (*p* < 0.05). The APC-fed asthmatic rats also showed a reduction in BT (of about 2.5 °C) with respect to their basal values in the period comprised between 0 and 16 h after the challenge (*p* < 0.01) and with respect to the REF animals’ BT in the period comprised between 4 and 16 h (*p* < 0.01). In the APC-fed group, the BT mean profile was the lowest (Figure 3a). Finally, the CMC-fed asthmatic rats also showed a decrease in BT (of about 2 °C) from immediately after the challenge until 10 h after, when it was compared to their basal values (*p* < 0.05), and during the period from 4 to 16 h after the challenge when compared to the REF animals’ BT (*p* < 0.05).

The effects of diets on BT in the first hours after handling have been considered as AUC (Figure 3b). It can be observed that the BT was the lowest in the asthmatic rats fed either a standard diet or APC diet (*p* < 0.01)

#### 3.3.4. Changes in Motor Activity After i.n. Challenge

**Figure 4:**
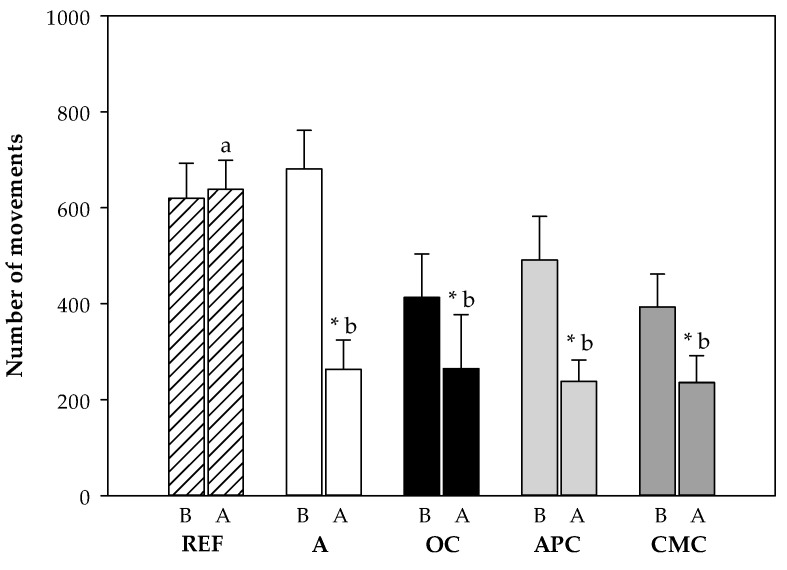
Number of movements during the active period (10 h of darkness) before (B) and after (A) intranasal challenge. REF: healthy animals fed standard diet; A: asthmatic animals fed standard diet; OC: asthmatic animals fed with ordinary cocoa-enriched diet; APC: asthmatic animals fed with “Amazonas Peru” cocoa-enriched diet; CMC: asthmatic animals fed with “Criollo de Montaña” cocoa-enriched diet. Results are shown as mean ± standard error (*N* = 9). * represents statistical differences from individual values before challenge (paired t-test) (*p* < 0.05). Values not sharing letters denote significant differences between groups (*p* < 0.01) while values sharing the same letter did not differ.

The motor activity (MA) of each rat was registered from the day before the i.n. challenge to 18 h after. To summarize the results and avoid the period in which animals were handled, the MA in the dark period (activity period for rats) during the night before the i.n. challenge was compared to the MA during the night after the challenge (Figure 4). The challenge significantly decreased the MA in all the asthmatic groups compared to the REF group (*p* < 0.05). In particular, it resulted in a decrease of MA by about 60% in the asthmatic animals fed the standard diet with respect to their own basal MA (*p* = 0.001). However, the cocoa samples-fed asthmatic rats exhibited a lower reduction (of about 40–50%) in the MA in comparison to their MA before the challenge (*p* < 0.05). The MA of cocoa-fed animals did not differ from that of the asthmatic rats fed a standard diet.

#### 3.3.5. Changes in Body Weight After i.n. Challenge

**Figure 5:**
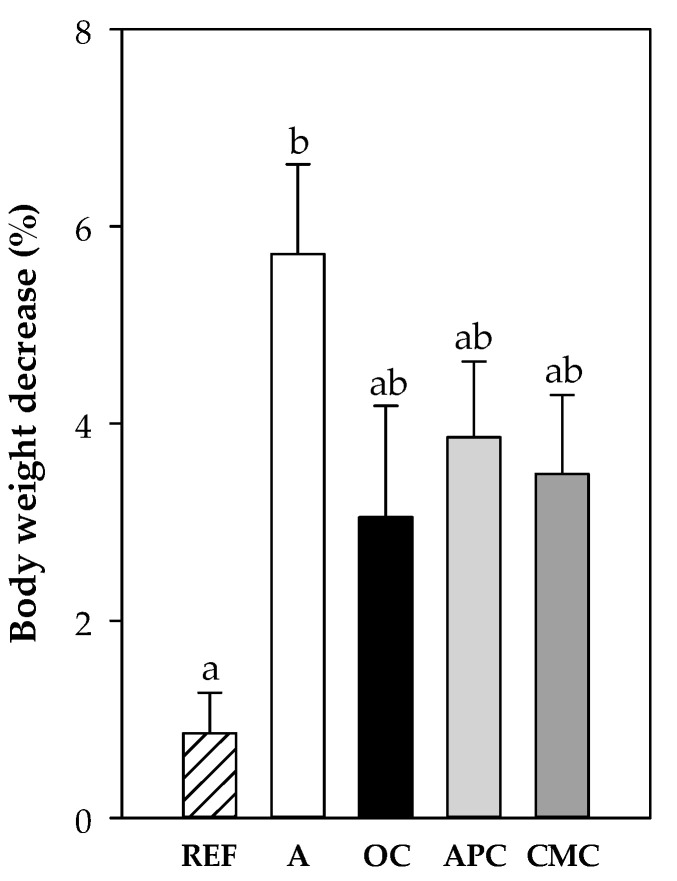
Body weight decrease (%) 24 h after the intranasal challenge with respect to the body weight before the challenge. REF: healthy reference group fed standard diet; A: asthmatic group fed standard diet; OC: asthmatic animals fed with ordinary cocoa-enriched diet; APC: asthmatic animals fed with “Amazonas Peru” cocoa-enriched diet; CMC: asthmatic animals fed with “Criollo de Montaña” cocoa-enriched diet. Results are represented as mean ± standard error of the mean (*N* = 9). Values not sharing letters denote significant differences between groups (*p* < 0.01), while values sharing the same letter did not differ.

One day after the i.n. challenge, all groups showed a significant decrease in body weight with respect to the body weight before the challenge (Figure 5). The asthmatic animals fed a standard diet decreased body weight by about 6%, whereas it decreased by up to 4% in cocoa-fed animals. Only the decrease in A group was significantly higher than that in the REF group (*p* < 0.001).

#### 3.3.6. Rat Mast Cell Protease II (RMCPII)

**Figure 6:**
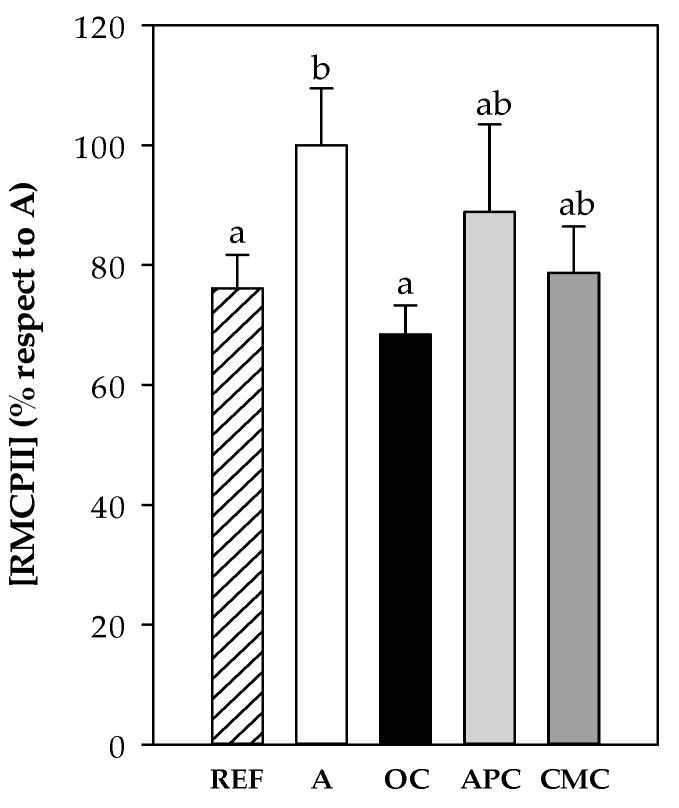
Rat mast cell protease II (RMCPII) in plasma obtained 1 h after the intranasal challenge. REF: healthy animals fed standard diet; A: asthmatic animals fed standard diet; OC: asthmatic animals fed with ordinary cocoa-enriched diet; APC: asthmatic animals fed with “Amazonas Peru” cocoa-enriched diet; CMC: asthmatic animals fed with “Criollo de Montaña” cocoa-enriched diet. Results are shown as mean ± standard error (*N* = 9). Results are expressed as mean ± standard error (*N* = 9) of absorbance units. Values not sharing letters denote significant differences between groups (*p* < 0.01), while values sharing the same letter did not differ.

RMCPII concentration was determined in plasma samples collected 1 h after the i.n. challenge from all experimental groups. The asthmatic group (A group) showed higher RMCPII concentration than that in the REF animals (Figure 6) (*p* < 0.01). Both the APC and CMC diets tended to prevent such increase whereas the OC diet was able to maintain the RMCPII values similar to those found in the REF group.

#### 3.3.7. IgE Antibodies

**Figure 7:**
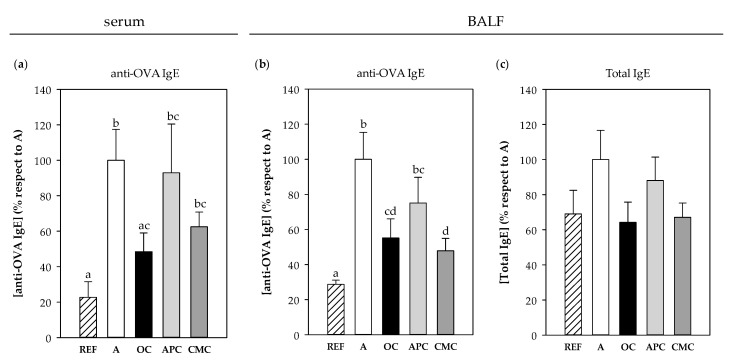
Anti-OVA IgE concentration in serum (**a**) and BALF (**b**) and total IgE content in BALF (**c**) obtained 24 h after the intranasal challenge. REF: healthy animals fed standard diet; A: asthmatic animals fed standard diet; OC: asthmatic animals fed with ordinary cocoa-enriched diet; APC: asthmatic animals fed with “Amazonas Peru” cocoa-enriched diet; CMC: asthmatic animals fed with “Criollo de Montaña” cocoa-enriched diet. Results are shown as mean ± standard error (*N* = 9). Values not sharing letters denote significant differences between groups (*p* < 0.01), while values sharing the same letter did not differ.

Specific anti-OVA IgE Ab concentration was quantified in serum and BALF samples obtained the day after the i.n. challenge. There were significant levels of specific IgE in the serum of asthmatic animals fed a standard diet (*p* < 0.05 vs. REF group) (Figure 7a). However, both OC and CMC diets were able to partially prevent such an increase, this reduction being significant only in the OC group, whose anti-OVA IgE levels were 50% lower than those in the A group (*p* = 0.021). In the BALF samples, both the OC and CMC groups showed a reduction of about 50% in the anti-OVA IgE levels in comparison to those observed in the asthmatic group (*p* < 0.05, Figure 7b). No significant changes were observed in the APC group in any of the sample types.

Total IgE content was also quantified in BALF, but in this case, although a similar profile to specific IgE was observed, no statistically significant differences were detected (Figure 7c).

There was a correlation between specific IgE levels in serum and BALF samples and the RMCPII content in plasma quantified in samples from 1 h after i.n. challenge (r = 0.370 *p* = 0.029 and r = 0.440 *p* = 0.008, respectively). However, no correlations were found between IgE levels and changes in MA and BT (data not shown).

#### 3.3.8. Leukotrienes

**Figure 8:**
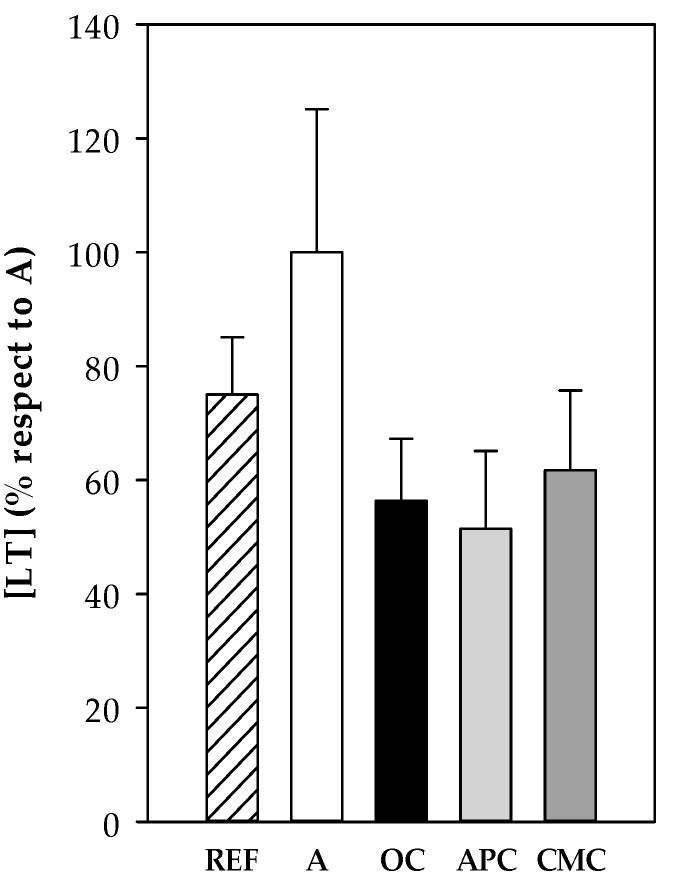
CysLT content in BALF samples obtained 24 h after the intranasal challenge. REF: healthy animals fed standard diet; A: asthmatic animals fed standard diet; OC: asthmatic animals fed with ordinary cocoa-enriched diet; APC: asthmatic animals fed with “Amazonas Peru” cocoa-enriched diet; CMC: asthmatic animals fed with “Criollo de Montaña” cocoa-enriched diet. Results are shown as mean ± standard error (*N* = 9).

Cys-LT concentration was determined in BALF samples collected 24 h after the i.n. challenge from all experimental groups. The asthmatic animals showed the highest values, but they did not reach statistical significance (Figure 8). This tendency to increase was partially reverted by all three diets containing cocoa samples.

No correlations were found between Cys-LT and RMCPII, IgE levels and changes in MA and BT.

## 4. Discussion

In the current study, the preventive potential of several Peruvian cocoa samples for allergic asthma has been approached. Cocoa has been considered beneficial for several chronic diseases [50,51,52,53], and it has been reported that such positive effects are mainly due to the composition of its bioactive compounds [54]. The content of such bioactive compounds in cocoa is largely dependent not only on the agricultural and postharvest practices and processing, but also on the cultivar and origins of cocoa [22,23]. In this sense, herein we have tested different Peruvian cocoa samples cultivated in various regions of the country, which could impact differently on human health due to their different bioactive compound contents. Therefore, the biochemical characterization along with the in vitro properties of four native Peruvian populations—all high quality cocoa used for making artisan chocolates—have been analyzed. At the same time, their properties were compared to an ordinary Peruvian cocoa but from an Ecuadorean clone [55]. It has been observed that the content of polyphenols, flavonoids and methylxanthines was distinct between the populations tested, thus confirming that environmental and genetic factors significantly influence their content, as previously reported [23]. The “Criollo de Montaña” (CMC) population was the richest in flavonoid (and polyphenol) concentration, as well as in methylxanthines content. This population grows in the Junín region (Satipo city) at 480 m.a.s.l. where the climate is predominantly hot and humid with a fluctuating temperature of between 18 and 35 °C. The second population with a higher content in flavonoids/polyphenols was the “Amazonas Peru” (APC) cocoa, which also exhibited the highest antioxidant activity, followed by the CMC population. The antioxidant properties of both the APC and CMC populations were also observed in the in vitro ROS production by macrophages, which was significantly decreased by these populations. Therefore, the higher the content of polyphenols these populations have, the stronger the antioxidant effects they exert, as would be expected.

The CCC (“Chuncho”) population exerted in vitro inhibitory effects on the inflammatory mediators’ secretion, such as TNF-α, by LPS-stimulated splenocytes, in agreement with previous in vitro studies using a conventional cocoa and particular cocoa flavonoids [13]. The anti-inflammatory properties of cocoa samples were also evidenced when analyzing the phenotype of in vitro LPS-stimulated macrophages. In this sense, the results obtained show that the native populations from BPC (“Blanco de Piura”) and APC had the potential to downregulate pro-inflammatory macrophage proportion while upregulating those from anti-inflammatory cells. Similar effects have been reported in the THP1 cell line cultured with a cocoa phenolic extract, which was able to induce a phenotypic switch in polarized macrophages in favor of the anti-inflammatory one [56].

On the other hand, all native populations tested in the present study, and also the ordinary cocoa, were able to downregulate the in vitro ability to produce immunoglobulins. This effect is in line with in vivo studies reporting a reduction of not only plasma IgG concentration, but also IgM and IgA in cocoa-fed animals [57,58]. This downregulation of immunoglobulin secretion seems to be due to an inhibitory B cell differentiation caused by the decrease in Th2 cytokines [59]. Overall, given that allergy is a Th2-associated response, these results encouraged us to evaluate the effects of cocoa in the present allergic asthma rat model.

As previously mentioned, based on their polyphenol content, their antioxidant activity and their in vitro effects, two cocoa populations (“Amazonas Peru” and “Criollo de Montaña”) were selected to be used in vivo in an allergic asthma model. In fact, previous studies have demonstrated the antiallergic properties of cocoa in preclinical studies, in models of systemic disease [18,19] and oral sensitization [20], and in observational studies considering cocoa consumption habits in young people with allergy [21].

Herein, body temperature and motor activity variables have been used to assess the anaphylactic response after i.n. challenge as previously set up [47] and also as used earlier in a model of oral allergy [19,48]. In addition, the decrease in body weight was also considered as a variable to assess anaphylaxis. Our results evidenced that the anaphylactic response was accompanied by a reduction in motor activity, which was not modified by any cocoa diet, although this decrease in cocoa-fed animals was relatively lower. Previous studies had reported a reduction in motor activity in animals fed with cocoa and receiving an oral challenge [19]. Nevertheless, when changes in body temperature were considered, some differences appeared. The asthmatic group fed a standard diet showed a significant decrease in body temperature that appeared earlier than the other groups and was more long-lasting, while the OC- and CMC-fed asthmatic animals maintained their body temperature similar to that found in the REF group for a longer time. This partial prevention in body temperature decrease by OC and CMC cocoa samples does not match with the vasodilator properties reported for cocoa [52] and the effects observed on a food allergy model [19]. On the other hand, the protective effects of cocoa samples were also observed in the decrease in body weight after i.n. challenge. Furthermore, the increase in plasma RMCPII released by activated mucosal mast cells [60] was partially prevented by both OC and CMC cocoas. In summary, the CMC population appears to be the native Peruvian population with the most potential to prevent anaphylactic response after i.n. challenge, and this ability is shared and even higher with the OC clone.

Diets containing Peruvian cocoa samples were able to differently influence the synthesis of specific IgE antibodies. Again, the CMC cocoa paste was the most effective native population at decreasing both the serum and bronchoalveolar lavage fluid IgE concentration, but its effects did not differ from the non-native OC cocoa. The protective effect of cocoa on IgE synthesis found here has already been described in other allergy models [8,18], and it confirms the immunomodulatory effects of a 10% cocoa diet in preclinical studies. Similarly, the anti-asthmatic effect of some extracts rich in flavonoids has also been demonstrated in an asthmatic animal model [61,62] and also in a limited number of clinical trials [63]. In agreement with these effects, the consumption of polyphenol-containing apple extracts was associated with an alleviation of some allergic symptoms, such as runny nose and nasal congestion in subjects suffering from allergic rhinitis [64].

Leukotrienes are other inflammatory molecules released when allergen binds to the mast cell-coupled IgE in allergy, which are primarily responsible for the bronchoconstriction during asthma attacks [60]. In our model, its concentration tended to increase in the asthmatic animals fed a standard diet, but all cocoa diets tended to prevent it and showed similar content as the reference group. All three cocoa samples tested in the present study contain theophylline, a methylxanthine naturally present in small amounts (1.40 mg/100 g) in cocoa beans that came out as a clinical treatment for asthma and other respiratory diseases once its bronchodilator effects had been identified [65]. In fact, it has been found that theophylline can act as suppressant of leukotriene production [66].

From the results obtained in in vivo experiments, it can be hypothesized that the main mechanism by which cocoa diets can exert a protective effect against allergic asthma response is by attenuating the synthesis of Th2-related antibodies. In particular, the cocoa diets have shown their anti-allergic potential mainly reducing the anti-OVA IgE levels. Therefore, the lower the IgE production is, the lower the amount of this antibody which can bind to mast cells in the airway. Consequently, when a new allergen contact is produced, the allergen may bind to only a little mast cell-bound IgE. For this reason, the release of mediators such as proteases and leukotrienes in the bronchoalveolar compartment is low and a weak anaphylactic response can be observed. The mechanisms produced by the effective cocoa populations must be addressed in further studies.

Polyphenol content in urine was also determined for approaching flavonoid absorption. The animals fed the CMC diet, which was elaborated with the native Peruvian population that exerted the most protective effects, had the highest polyphenol concentration in urine. Nevertheless, the OC diet also showed a protective response against anaphylactic response and showed the lowest urine polyphenol concentration. Therefore, it is not just the flavonoid content that seems to be important in playing a protective role in this model, but it may also be the type of flavonoids (for example monomers or polymeric forms) present in each population. Anyway, as CMC has the most immunomodulatory properties of the Peruvian “fine aroma” cocoa populations evaluated in this study, further experiments focused on particular cocoa flavonoids or other cocoa bioactive compounds in this population must shed light on this issue.

The cocoa used as raw material for chocolate is a source of differences in terms of its sensorial quality, as can be observed between ordinary cocoa and fine aroma cocoa. Ordinary cocoa has the most widespread use in the industry, being used for common chocolates, while fine aroma cocoa is used for fine or artisan chocolates. The result of this study shows that the origin of the cocoa populations from which the chocolate pastes are obtained determines differences that are expressed at the biochemical level and in their bioactivity. Chocolate pastes, made with cocoa taken from different populations in the same country (Peru), are biochemically differentiable and have different bioactivity, as demonstrated in the present study.

## 5. Conclusions

Overall, it has been shown that particular populations of Peruvian cocoa exhibit a protective effect on a rat model of acute allergic asthma response. This effect can be observed by means of a partial protection against anaphylactic response and, above all, in the synthesis of IgE and the release of mast cell protease. These results show that the origin of cocoa is relevant and should be taken into account and declared in these types of studies, and probably also when it comes to be used in the making of dark chocolate or as nutraceuticals.

## Figures and Tables

**Table 1:** Content of total polyphenols and flavonoids, methylxanthines and antioxidant capacity in the cocoa populations considered in the study. APC: “Amazonas Peru” cocoa; BPC: “Blanco de Piura” cocoa; CCC: “Chuncho” cocoa; CMC: “Criollo de Montaña” cocoa; OC: ordinary cocoa. Results are represented as mean ± standard error of the mean from three independent experiments. Values not sharing letters denote significant differences between populations (*p* < 0.01) while values sharing the same letter did not differ.

	OC	BPC	APC	CMC	CCC
**Total phenolics** (mg gallic acid equivalents/g)	24.11 ± 1.15 ^ab^	21.07 ± 0.98 ^a^	28.69 ± 0.80 ^c^	30.44 ± 0.56 ^d^	25.30 ± 0.38 ^b^
**Total flavonoids** (mg catechin equivalents/g)	34.82 ± 1.01 ^a^	36.59 ± 0.98 ^a^	50.35 ± 0.95 ^b^	56.62 ± 1.12 ^c^	45.60 ± 1.26 ^d^
**Theobromine** (mg/100 g)	560.75 ± 0.45 ^a^	491.39 ± 0.93 ^b^	564.71 ± 0.37 ^a^	604.19 ± 0.28 ^d^	573.36 ± 0.30 ^e^
**Theophylline** (mg/100 g)	1.41 ± 0.00 ^a^	1.37 ± 0.00 ^b^	1.40 ± 0.00 ^c^	1.39 ± 0.00 ^d^	1.54 ± 0.00 ^e^
**Caffeine** (mg/100 g)	236.35 ± 0.06 ^a^	280.74 ± 0.56 ^b^	275.10 ± 0.11^c^	360.53 ± 0.27 ^d^	324.55 ± 0.15 ^e^
**FRAP activity** (µmol Fe^2+^/g)	275.29 ± 12.28 ^ab^	261.39 ± 14.15 ^a^	344.31 ± 11.80 ^c^	309.55 ± 6.7 ^b^	308.32 ± 4.79 ^b^
**DPPH activity** (µg TEAC/g)	25.36 ± 0.24 ^ab^	19.62 ± 0.38 ^c^	29.77 ± 0.70 ^a^	25.38 ± 0.94 ^bd^	24.39 ± 0.07 ^d^

**Table 2:** Body weight for all experimental groups throughout the study. REF: healthy reference group fed standard diet; A: asthmatic group fed standard diet; OC: asthmatic group fed 10% ordinary Peruvian cocoa; APC: asthmatic group fed 10% “Amazonas Peru” cocoa; CMC: asthmatic group fed 10% “Criollo de Montaña” cocoa. Results are represented as mean ± standard error of the mean (*N* = 9).

Time (days)	REF	A	OC	APC	CMC
**−7**	57.01 ± 5.04	57.52 ± 3.32	56.43 ± 3.59	56.46 ± 3.54	56.97 ± 2.63
**−3**	67.38 ± 5.37	68.60 ± 3.57	65.37 ± 3.86	64.84 ± 3.80	64.03 ± 2.90
**0 ^a^**	75.96 ± 5.37	76.97 ± 3.55	72.44 ± 3.79	72.66 ± 4.03	71.91 ± 2.79
**4**	85.07 ± 4.80	84.76 ± 3.10	79.31 ± 3.65	79.21 ± 3.80	78.47 ± 2.59
**7 ^b^**	91.57 ± 4.66	91.33 ± 3.57	82.73 ± 3.50	85.10 ± 3.94	84.48 ± 2.72
**11**	98.29 ± 5.52	93.70 ± 3.76	88.29 ± 3.34	89.31 ± 3.99	89.43 ± 2.67
**14**	104.03 ± 4.66	99.94 ± 3.74	94.59 ± 4.16	94.96 ± 4.28	94.07 ± 2.82
**18**	109.49 ± 4.88	105.90 ± 3.62	97.99 ± 4.68	97.98 ± 4.15	99.27 ± 2.92
**21**	115.80 ± 4.86	112.00 ± 3.72	104.26 ± 4.58	104.24 ± 4.49	105.77 ± 2.89
**25**	118.29 ± 5.58	115.68 ± 3.62	107.66 ± 4.93	108.10 ± 4.39	107.65 ± 2.52
**28**	123.73 ± 5.16	118.09 ± 2.46	109.46 ± 4.52	111.95 ± 4.03	112.97 ± 2.71

^a^ day of sensitization; ^b^ day of booster.

**Table 3:** Temperature means at 2 h intervals from 2 h before until 18 h after the intranasal challenge. REF: healthy animals fed standard diet; A: asthmatic animals fed standard diet; OC: asthmatic animals fed with ordinary cocoa-enriched diet; APC: asthmatic animals fed with “Amazonas Peru” cocoa-enriched diet; CMC: asthmatic animals fed with “Criollo de Montaña” cocoa-enriched diet. Results are represented as mean ± standard error of the mean (*N* = 9).

Time (h)	REF	A	OC	APC	CMC
**−2 to 0**	37.7 ± 0.11	37.6 ± 0.10	37.8 ± 0.18	37.9 ± 0.18	37.7 ± 0.13
**0 to 2**	37.1 ± 0.61	36.7 ± 0.29 *	37.1 ± 0.19	36.1 ± 0.44 *	36.2 ± 0.33 *
**2 to 4**	36.7 ± 0.51 *^,a^	35.1 ± 0.58 *^,b^	36.2 ± 0.24 *^,a^	35.3 ± 0.68 *^,a^	35.8 ± 0.32 *^,a^
**4 to 6**	37.5 ± 0.14 *^,a^	35.5 ± 0.41 *^,b^	36.2 ± 0.37 *^,b^	35.6 ± 0.44 *^,b^	36.1 ± 0.43 *^,b^
**6 to 8**	37.7 ± 0.11 ^a^	36.2 ± 0.23 *^,b^	36.5 ± 0.44 *^,b^	36.1 ± 0.28 *^,b^	36.3 ± 0.39 *^,b^
**8 to 10**	37.9 ± 0.11 ^a^	36.6 ± 0.17 *^,b^	36.4 ± 0.58 ^b^	36.4 ± 0.36 *^,b^	36.7 ± 0.37 *^,b^
**10 to 12**	38.0 ± 0.09 ^a^	36.9 ± 0.18 *^,b^	36.9 ± 0.50 ^a^	36.5 ± 0.40 *^,b^	36.8 ± 0.39 ^b^
**12 to 14**	37.9 ± 0.12 ^a^	37.1 ± 0.18 *^,b^	37.0 ± 0.52 ^a^	36.8 ± 0.30 *^,b^	36.9 ± 0.38 ^b^
**14 to 16**	38.1 ± 0.09 ^a^	37.2 ± 0.12 *^,b^	37.1 ± 0.60 ^a^	37.2 ± 0.25 *^,b^	37.0 ± 0.40 ^b^
**16 to 18**	38.0 ± 0.14 ^a^	37.3 ± 0.18 *^,b^	37.2 ± 0.64 ^a^	37.4 ± 0.24 ^a^	37.4 ± 0.27 ^a^

* represents statistical differences vs. their own basal values before challenge (−2 to 0) (*p* < 0.05). Values not sharing letters denote significant differences between groups during the same period of time (*p* < 0.01) while values sharing the same letter did not differ.

## Supplementary Materials

The following are available online at https://www.mdpi.com/2072-6643/12/8/2301/s1, Table S1: Food intake for all experimental groups throughout the study. Table S2: Water consumption for all experimental groups throughout the study.
