# Supplementary material for: Attenuating Effect of Peruvian Cocoa Populations on the Acute Asthmatic Response in Brown Norway Rats
Source: Nutrients. 2020 Jul 31;12(8):2301. doi: 10.3390/nu12082301 (PMC7469048; doi:10.3390/nu12082301)
Supplement: Supplementary file 1 [file nutrients-12-02301-s001.zip › Supplementary Materials - Table S1 Periz et al.docx]

**Supplementary Materials**

**Table S1**

| **Time (days)** | **REF** | **A** | **OC** | **APC** | **CMC** |
| --- | --- | --- | --- | --- | --- |
| **-3** | 12.90 ± 0.155 | 12.45 ± 0.161 | 12.97 ± 0.248 | 12.14 ± 0.382 | 15.00 ± 2.337 |
| **0^a^** | 11.79 ± 0.195 | 11.52 ± 0.156 | 12.02 ± 0.181 | 12.11 ± 0.184 | 14.48 ± 1.917 |
| **4** | 10.76 ± 0.786 | 10.99 ± 0.445 | 11.86 ± 0.354 | 11.23 ± 0.740 | 13.40 ± 1.221 |
| **7^b^** | 9.83 ± 0.611 | 9.76 ± 0.231 | 10.53 ± 0.996 | 10.43 ± 0.440 | 12.43 ± 1.340 |
| **11** | 8.05 ± 1.301 | 8.28 ± 0.218 | 8.90 ± 0.610 | 9.10 ± 0.466 | 11.19 ± 1.173 |
| **14** | 8.22 ± 0.483 | 8.65 ± 0.857 | 9.04 ± 0.128 | 8.30 ± 0.735 | 10.11 ± 1.164 |
| **18** | 6.45 ± 1.107 | 6.78 ± 1.205 | 6.43 ± 1.269 | 6.41 ± 0.734 | 9.53 ± 0.904 |
| **23** | 6.48 ± 0.424 | 7.10 ± 0.172 | 7.13 ± 0.238 | 6.69 ± 0.471 | 7.33 ± 0.405 |
| **25** | 7.87 ± 0.311 | 7.00 ± 0.781 | 8.11 ± 0.297 | 8.06 ± 0.695 | 8.80 ± 0.833 |
| **28** | 7.82 ± 0.139 | 6.98 ± 0.687 | 8.15 ± 0.222 | 7.83 ± 0.624 | 6.90 ± 0.931 |

^a^ day of sensitization; ^b^ day of booster. REF: healthy reference group fed standard diet; A: asthmatic group fed standard diet; OC: asthmatic group fed 10% ordinary Peruvian cocoa; APC: asthmatic group fed 10% “Amazonas Peru” cocoa; CMC: asthmatic group fed 10% “Criollo de Montaña” cocoa. Results are represented as mean ± standard error of the mean (N=3-9).
