# Supplementary material for: Attenuating Effect of Peruvian Cocoa Populations on the Acute Asthmatic Response in Brown Norway Rats
Source: Nutrients. 2020 Jul 31;12(8):2301. doi: 10.3390/nu12082301 (PMC7469048; doi:10.3390/nu12082301)
Supplement: Supplementary file 1 [file nutrients-12-02301-s001.zip › Supplementary Materials - Table S2 Periz et al.docx]

**Supplementary Materials**

**Table S2**

| **Time (days)** | **REF** | **A** | **OC** | **APC** | **CMC** |
| --- | --- | --- | --- | --- | --- |
| **-3** | 10.84 ± 1.090 | 10.80 ± 1.425 | 14.02 ± 0.506 | 13.62 ± 1.753 | 12.52 ± 3.549 |
| **0^a^** | 10.97 ± 1.304 | 10.45 ± 1.486 | 15.02 ± 1.350 | 15.36 ± 1.052 | 15.39 ± 3.941 |
| **4** | 11.03 ± 2.563 | 8.46 ± 1.457 | 9.81 ± 0.633 | 13.23 ± 3.051 | 13.11 ± 3.822 |
| **7^b^** | 9.69 ± 1.996 | 9.07 ± 1.457 | 10.50 ± 0.195 | 10.00 ± 0.152 | 13.83 ± 4.503 |
| **11** | 7.44 ± 0.322 | 5.66 ± 1.068 | 9.93 ± 0.273 | 7.72 ± 0.722 | 9.42 ± 1.120 |
| **14** | 6.78 ± 0.376 | 5.73 ± 0.249 | 8.82 ± 1.093 | 7.60 ± 0.989 | 9.12 ± 1.091 |
| **18** | 6.57 ± 1.265 | 5.96 ± 0.880 | 7.24 ± 1.378 | 5.81 ± 0.980 | 7.96 ± 1.329 |
| **23** | 7.23 ± 0.373 | 7.97 ± 0.319 | 9.76 ± 0.839* | 8.81 ± 0.479* | 8.32 ± 0.636 |
| **25** | 7.30 ± 0.966 | 8.63 ± 1.222 | 9.24 ± 1.273 | 7.93 ± 0.711 | 6.18 ± 0.736 |
| **28** | 8.81 ± 0.414 | 8.91 ± 1.857 | 8.53 ± 1.008 | 8.13 ± 1.010 | 7.54 ± 0.636 |

^a^ day of sensitization; ^b^ day of booster. REF: healthy reference group fed standard diet; A: asthmatic group fed standard diet; OC: asthmatic group fed 10% ordinary Peruvian cocoa; APC: asthmatic group fed 10% “Amazonas Peru” cocoa; CMC: asthmatic group fed 10% Criollo de Montaña cocoa. Results are represented as mean ± standard error of the mean (N=3-9).
